# Emerging Approaches to Mitigate Neural Cell Degeneration with Nanoparticles-Enhanced Polyelectrolyte Systems

**DOI:** 10.3390/membranes15100313

**Published:** 2025-10-14

**Authors:** Angelika Kwiatkowska, Anna Grzeczkowicz, Agata Lipko, Beata Kazimierczak, Ludomira H. Granicka

**Affiliations:** Nalecz Institute of Biocybernetics and Biomedical Engineering, Polish Academy of Sciences, Trojdena 4 st., 02-109 Warsaw, Poland; ankwiatkowkska@op.pl (A.K.); agrzeczkowicz@ibib.waw.pl (A.G.); alipko@ibib.waw.pl (A.L.);

**Keywords:** polyelectrolyte membrane coating, neurodegenerative diseases, neural cells, nanoparticles

## Abstract

Counteracting neurodegenerative diseases (NDs) presents a multifaceted challenge in the aging societies of Western countries. Each year, millions of people worldwide are affected by such ailments as Parkinson’s disease (PD), Alzheimer’s disease (AD), Huntington’s disease (HD), multiple sclerosis (MS), spinal cord injury, ischemic stroke, motor neuron disease, spinal muscular atrophy, spinocerebellar ataxia, and amyotrophic lateral sclerosis (ALS). Advancements in modern biomaterial technologies present substantial opportunities for the field of regenerative medicine. Nevertheless, limitations arise from the requirement that biomaterial design be tailored to the specific biological parameters of the target cell types with which they are intended to interact. Such an opportunity creates nanomaterials involving nanoparticles. The surface chemistry of nanoparticles, especially when functionalized with bioactive agents, enhances biocompatibility and facilitates interactions with nervous cells. Herein, we review contemporary strategies in the application of biomaterials for nerve regeneration, with particular emphasis on nanomaterials and biocompatible polyelectrolyte layers, which the authors identify as having the most significant potential to drive transformative advances in regenerative medicine in the near future.

## 1. Introduction

Counteracting neurodegenerative diseases (NDs) presents a multifaceted challenge in the aging societies of Western countries. Each year, millions of people worldwide are affected by such ailments as Parkinson’s disease (PD), Alzheimer’s disease (AD); Huntington’s disease (HD), multiple sclerosis (MS), spinal cord injury, ischemic stroke, motor neuron disease, spinal muscular atrophy, spinocerebellar ataxia, and amyotrophic lateral sclerosis (ALS) [1,2,3,4]. A few years ago, it was estimated that approximately 15% of the Earth’s population might suffer from neurodegenerative diseases [5,6]. In 2019 alone, NDs caused the death of 10 million individuals, whereas 349.2 million people were afflicted [7]. The latest reports are even more pessimistic; a study published in *The Lancet Neurology* shows that as many as 3.4 billion individuals could live with a neurological condition in 2021 [8]. Such a situation leaves a deep economic footprint as developed countries spend vast amounts of money on programs supporting the prevention and treatment of diseases of this type [5,9]. As a study from 2022 states, the total annual per capita cost of Alzheimer’s disease alone ranged from US$468.28 in mild cases to US$171,283.80 in severe cases, with care-related expenses increasing nonlinearly with disease progression [10]. According to the report, including 93% of the world’s population, Alzheimer’s disease and other dementias are projected to impose an economic burden of $14,513 billion in international dollars from 2020 to 2050, which corresponds to an average of 0.421% of the annual global gross domestic product (GDP) [11].

**Figure 1:**
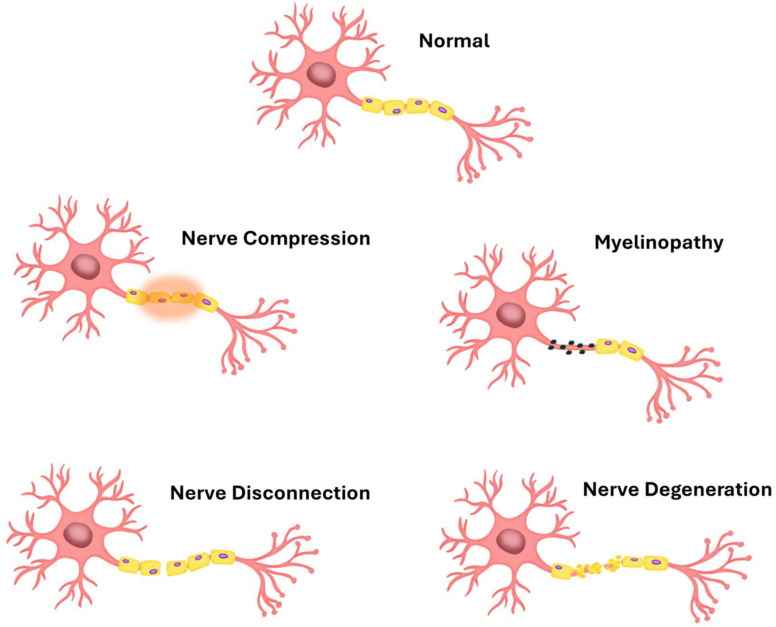
Nerve degeneration according to Waller.

Neurodegenerative diseases are characterized by progressive damage to the nervous system’s structures. These disorders are currently essentially incurable, as mature cells of the nervous system do not tend to regenerate. In Figure 1, a schematic representation of nervous cell degeneration, as described by Waller, is presented.

Moreover, in addition to NDs, nerve damage can also occur as a result of traumatic brain injury, spinal cord injury, and peripheral nerve injury [12]. Nevertheless, regardless of the cause and type of damage, in each case, it is necessary to use regenerative medicine to restore the original, lost functions of nerve cells.

Neural tissue is the primary component of the nervous system and is found in the brain, spinal cord, and nerves. It is composed of two types of cells: neurons and neuroglial cells. There are three types of the latter: astrocytes, oligodendrocytes, and microglia, which are supposed to play a supporting role in neurons’ activity. Moreover, they form myelin and provide neurons with nutrition. Neurons, the basic units of nervous tissue, generate and conduct impulses, which are responsible for cell-to-cell communication. The cells are composed of a soma, which includes the nucleus and other functional organelles, dendrites that receive signals from other neurons, and axons that receive signals from the cell body and transmit them to the axon terminals, where neurotransmitters are released to connect to muscles or glands. The dysfunction of neurons can be caused by neurodegeneration, resulting from the progressive loss of neurons and/or their functions, which is associated with dysfunction of the synapse and neural network [12,13].

**Figure 2:**
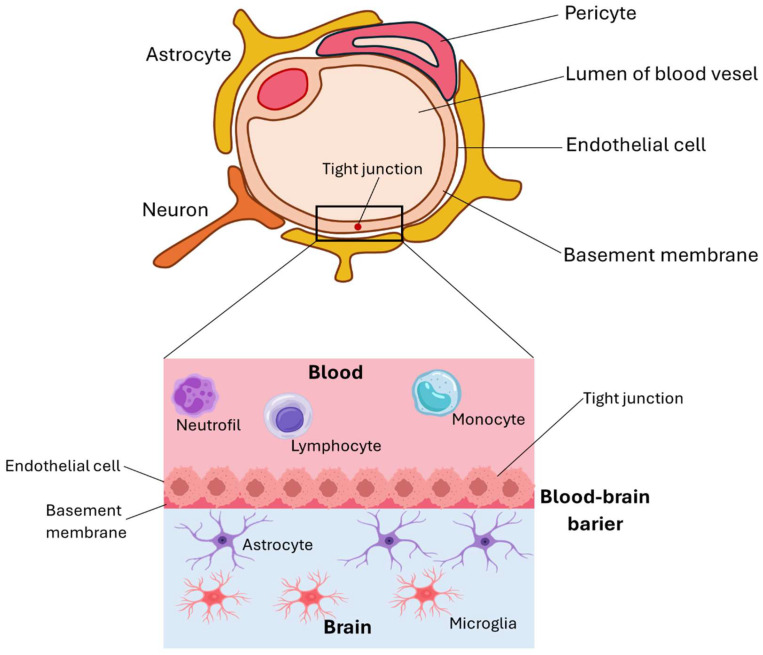
Blood–brain barrier.

As mentioned above, nervous tissue is characterized by poor regenerative capacity; therefore, all approaches aimed at supporting its growth are under extensive evaluation. For example, neural stem cell-based therapies for central nervous system restoration have attracted growing interest recently due to their potential for reconstructing damaged tissue through exogenous neural cell transplantation. The reason for this is that neural stem cells proliferate and are capable of long-term self-renewal, unlike mature neurons, which do not proliferate. Alternatively, some researchers place greater emphasis on advancements in materials engineering, proposing that the development of novel biomaterial surfaces capable of supporting neural regeneration may offer scalable and economically advantageous solutions for tissue repair. It may, in turn, enable the use of techniques from the field of nanotechnology and neurobiology, providing the opportunity to, among other things, thoroughly understand the structure and function of nervous tissue and influence the function of cells cultured on constructed substrates. Biomaterials engineering addresses nerve growth inhibition and loss of long-distance control problems, allowing for the expansion of therapy beyond cell-based approaches. Research has been conducted on materials that exhibit electrical and/or biomechanical properties, which trigger and enhance nerve regeneration. For that purpose, natural, synthetic, and conductive polymers are considered. Materials with incorporated nanoparticles are also employed; they are particularly valued for their ability to cross the blood–brain barrier (Figure 2 illustrates this barrier). Moreover, biomaterial scaffolds in combination with growth factors, such as brain-derived neurotrophic factor (BDNF), basic fibroblast growth factor (bFGF), or vascular endothelial growth factor (VEGF), are reported to potentially stimulate neurogenesis directly at the injured site [14].

Herein, we review contemporary strategies in the application of biomaterials for nerve regeneration, with particular emphasis on nanomaterials and biocompatible polyelectrolyte layers, which the authors identify as having the most significant potential to drive transformative advances in regenerative medicine in the near future.

## 2. Trouble in Paradise—Short Overview of the Neuronal Cell Cultures

Regarding the aspect of cell culture, it is worth noting that neuronal cell culture provides a basis for studying the functioning of the nervous system. The fact that mature neurons do not undergo cell division encourages the use of immortalized secondary cell lines derived from neuronal tumors. Although an unlimited number of these cells can be obtained, they show physiological differences from the cell type from which they originate. These lines can be induced to exhibit a more neuronal phenotype by modifying the culture conditions, e.g., by adding specific growth factors. Among these lines, in which the expression of neuronal markers can be induced, we can mention the human neuroblastoma SH-SY5Y cell line, NTera, a human neuronally committed teratocarcinoma cell line, and PC12, a pheochromocytoma of the adrenal medulla derived rat cell line. Also used as models for studying neuronal cells are mouse embryonic stem cells, as well as the F9 and P19 cell lines.

**Figure 3:**
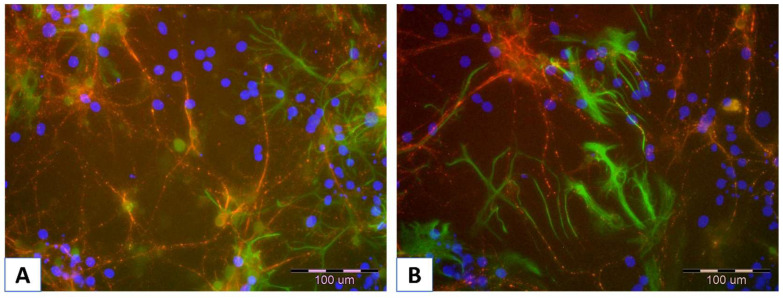
(**A**,**B**) Visualization of murine neuronal cells immobilized on alginate/chitosan polyelectrolyte bilayer after two weeks of culture. The red fluorescence shows neurons stained with anti-microtubule-associated protein 2 (MAP2), using a secondary antibody conjugated with the fluorochrome Alexa Fluor 555. The light green fluorescence indicates astrocytes stained with anti-glial fibrillary acidic protein (GFAP) and a secondary conjugated antibody labeled with Alexa Fluor 488 fluorochrome. Nuclei were dyed blue with Hoechst 33342 to enhance contrast under microscopy. Photos were taken using an Olympus IX71 (Olympus, Tokyo, Japan) fluorescence microscope.

Nevertheless, the chance to repeat the properties of neuronal cells in vivo is provided by the use of primary cell cultures, not derived from tumors. However, in this case, it is necessary to separate neuronal cells from other types of cells, such as astrocytes and oligodendrocytes [15]. In Figure 3A,B, the exemplary pictures of murine neuronal cells isolated from the embryonic brain, immobilized on an alginate/chitosan polyelectrolyte bilayer, are presented.

Neural cells (both isolated and cell lines) are characterized by poor adhesion to the surface of culture vessels. Their culture is commonly performed on substrate surfaces, such as those produced by poly-l-lysine (PLL) and poly-d-lysine (PDL), which are applied as monolayers [16,17,18,19]. Nonetheless, studies have been conducted to investigate the interaction of polyelectrolytes and their combinations with neural cells.

## 3. Nanomaterials in Nerve Regeneration

It has long been recognized that nanoparticles, which represent a type of nanomaterial, have revolutionized medical science, offering new horizons in regenerative therapies, particularly at the cellular level [20]. The benefits of using nanoelements go far beyond structural reinforcement. Nanoparticles’ unique size affects their diffusion properties, enabling them to penetrate tissues and consequently influence cellular uptake, which is vital for regeneration. On the other hand, the surface chemistry of nanoparticles, especially when functionalized with bioactive agents, enhances biocompatibility and facilitates interactions with nervous cells. Surface charge also plays a crucial role in determining how cells interact with and internalize nanoparticles. Therefore, understanding how surface charge, chemistry, and size affect biological responses is essential to optimizing nanoparticle-based nerve regeneration therapies.

**Figure 4:**
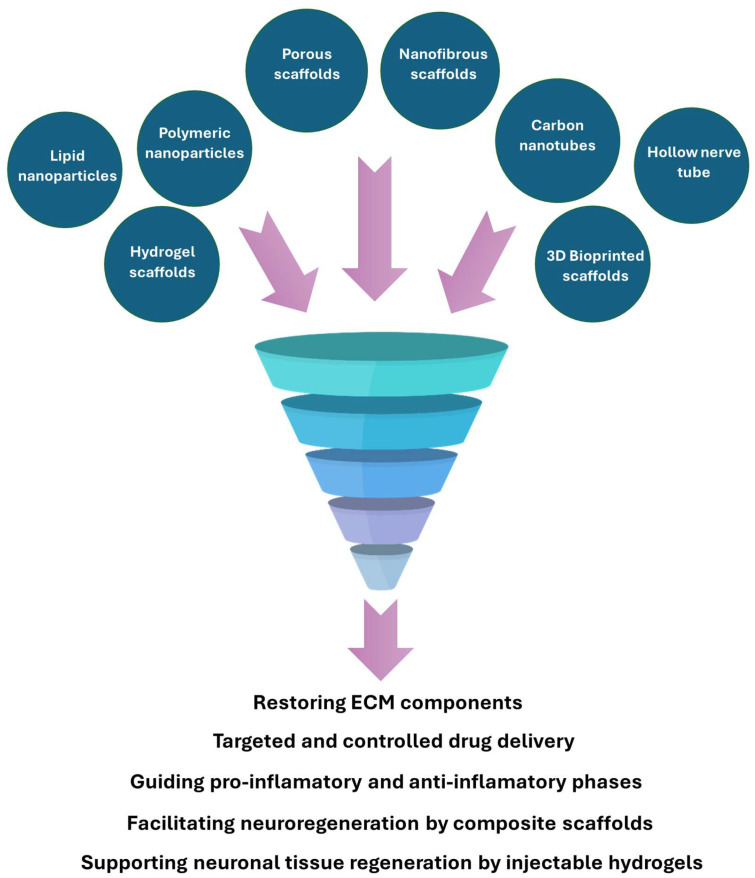
Different examples of biomaterial applications in nerve regeneration.

Additionally, the constantly increasing variety of available nanomaterials broadens the range of their possible usage in nerve regeneration therapies [21,22]. Figure 4 presents an overview of various examples of nanomaterials’ share among biomaterials applied in nerve regeneration.

One way to use nanoparticles is as carriers of active substances (like growth factors [23], neurotrophic agents [24], or genes [25,26]) in targeted therapies or as support facilitating regenerated tissue growth. Liposomes [27,28,29,30,31] and dendrimers [26,32,33,34] are excellent examples of nanoparticles designed for targeted drug delivery. On the other hand, nanofibers [8,35,36,37,38], nanowires [39,40,41,42,43], and graphene derivatives [44,45,46,47,48] serve as structures that mimic the extracellular matrix to support nerve cell growth. Furthermore, systems based on the antibacterial properties of nanoparticles are often developed to limit inflammatory reactions [49,50,51]. A different approach employs nanoparticles in theranostics [52,53,54,55]; fluorescent quantum dots are commonly used to enable imaging and tracking [56,57,58,59]. However, scientists have become cautious about the involvement of nanoparticles in nerve regeneration due to their potential toxicity [60,61,62,63], the risk of crossing the blood–brain barrier [60,64,65,66], and the accumulation of nanoparticles in organs following prolonged exposure [67,68,69,70,71,72].

### 3.1. The Influence of Nanomaterial Structure and Its Role in Neuroregeneration

The material’s structure, characteristics, and toxicological profile determine the diverse functionalities of nanoparticles in neuroregeneration. For example, the specificity of metal nanoparticles (such as gold, silver, and iron) makes them suitable for nerve regeneration and stimulation, as they possess enhanced electrical conductivity that mimics natural conditions [73,74,75]. In contrast, polymeric nanoparticles, characterized by above-average biocompatibility, are excellent carriers for therapeutic agents as they allow precise and controlled drug release, supporting neuroprotection and modulation of neural growth simultaneously [76,77,78,79]. The separate class of materials is hybrid nanoparticles that combine metallic and polymeric elements to harness multiple functionalities. Such an amalgamate enables electrical conductivity and ensures direct delivery mechanisms, enhancing therapeutic efficacy in nerve renewal [80]. Combined metallic and polyelectrolyte elements, e.g., magnetic alginate microparticles obtained by water-in-oil emulsion crosslinking of sodium alginate and iron oxide nanoparticle mixture, can be applied as a sacrificial form of tubular structure to template construction composed of glycidyl methacrylate, hyaluronic acid, and collagen I to replicate the microarchitecture of the native nerve basal lamina [81].

Another critical aspect of nanoparticles’ characteristics that should be considered before their application in nerve regeneration therapies is toxicity, as it influences the material’s interaction with biological systems [82,83]. Material toxicity is a derivative of its composition, particle size, and surface properties [84,85,86]. Therefore, providing biocompatibility and minimizing potential adverse effects of applied nanoelements requires considering their physicochemical profile, which, in turn, is often determined by the chosen synthesis method. Two main strategies are applied to receive nanoparticles: bottom-up and top-down, each offering unique advantages for biomedical applications [87,88]. In the bottom-up approach, nanoparticles are constructed from the atomic or molecular level, allowing for precise control over their nature and simplified surface functionalization (functional groups, proteins, peptides, or antibodies can be attached to the surface to improve internalization, cellular uptake, and/or biocompatibility) [87,88,89]. Moreover, such modifications enable scientists to obtain stimuli-responsive materials, allowing for the precise release of drugs in response to environmental triggers, such as temperature or pH [90,91,92]. Among the bottom-up synthesis techniques used to obtain materials for biomedicine, chemical synthesis, emulsification, sol–gel methods, self-assembly layering, thin-film hydration, and chemical, physical, electrochemical, or atomic layer deposition can be listed [90,93]. For instance, gold and silver nanoparticles obtained by chemical synthesis have unique optical and electrical properties, which are beneficial for imaging and targeted delivery of therapeutic agents in nerve regeneration [94]. On the other hand, thin-film hydration of lipid-based nanoparticles provides carriers for bioactive molecules, facilitating their transport across the blood–brain barrier to support nerve reconstruction [93]. Finally, polymeric carriers of bioactive molecules, which enhance their transport across the blood–brain barrier while promoting cell growth, can be obtained by applying self-assembly or emulsion polymerization methods [95,96,97,98].

On the contrary, top-down synthesis techniques involve deconstructing bulk materials into nanoparticles by etching or managed fragmentation, offering scalability and precise size control. Examples include laser and electroablation, mechanical grinding (such as ball milling), sputtering, etching, and lithography, which are commonly used to fabricate nanoparticles with specific dimensions and shapes [99]. It is worth noting that top-down approaches are beneficial for creating nanoparticles with tailored physical properties, which are suitable for integration into scaffolds ideal for nerve reconstruction [100].

### 3.2. Multifaceted Approach to Nerve Regeneration Using Nanomaterials

Nanomaterials offer a multifaceted approach to nerve restoration by promoting axonal growth, supporting myelination, mitigating oxidative stress, modulating inflammation, and enhancing neurotrophic factors, which positions them as a key tool for advancing cell regeneration therapies.

#### 3.2.1. NPs Mitigating Oxidative Stress and Modulating Inflammation

Nanoparticles’ ability to scavenge reactive oxygen species (ROS) and simultaneously reduce inflammation is commonly employed in nerve repair after spinal cord injuries (SCI) [101,102,103,104]. In general, excessive ROS production stimulates macrophage activation, engendering inflammation and intensifying the development of SCI. It is worth noting that macrophages in SCI are categorized into two subpopulations: M1 (proinflammatory) and M2 (anti-inflammatory). A shift between M1 and M2 polarization alters the balance of inflammation, influencing the progression and recovery of the injury as different cytokine types are released. Nanoparticles are used to regulate the balance of macrophages [105]. For instance, it has been shown that selenium nanoparticles (SeNPs) derived from *Proteus mirabilis* YC801 reduce M1 macrophages by suppressing proinflammatory factors (TNF-α, IL-1β) and increase M2 macrophages by elevating anti-inflammatory cytokines (IL-4, IL-10), thereby modulating microglial responses to promote nerve recovery in rats with spinal cord injury [102]. A further example is curcumin/poly(−)-epigallocatechin-3-gallate-encapsulated nanoparticles (HA-CurNPs), which promote anti-inflammatory M2 microglial polarization and suppress proinflammatory M1 polarization by inhibiting CD74 expression. Studies have shown that such modulation reduces inflammation, facilitates neuronal repair, and supports motor function recovery in mice with spinal cord injury (SCI) [103]. In turn, scientists have demonstrated that mannose-coated magnetic nanoparticles (mSPIONs) reduce proinflammatory cytokines (IL-6 and TNF-α), thereby creating a conducive environment for nerve regeneration [106]. Other examples of nanomaterials’ usage in mitigating oxidative stress and modulating inflammation are presented in Table 1.

#### 3.2.2. Remyelination, Axonal Growth, and NPs for Its Promotion

**Figure 5:**
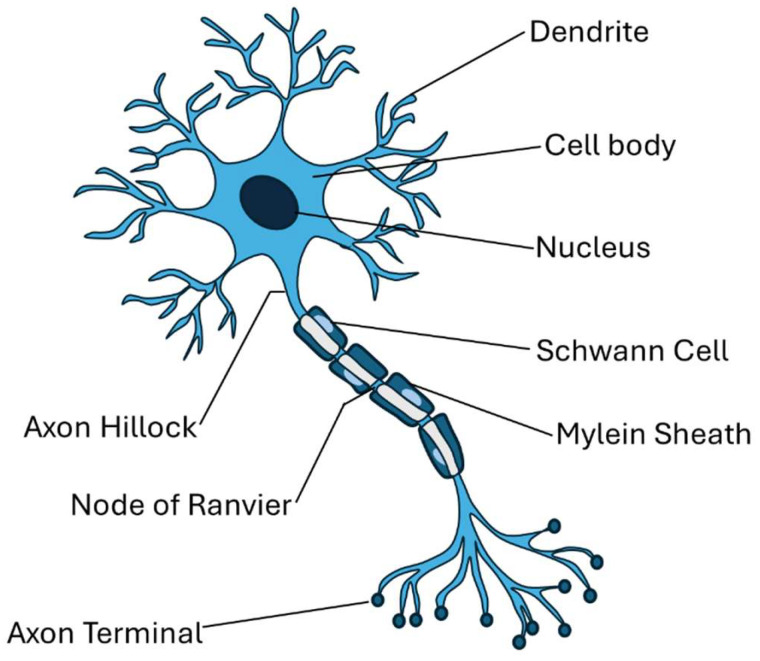
Neuron structure with myelin sheath and Schwann cells.

The promotion of remyelination and axonal growth after injury is an additional example of the beneficial effects of nanoparticles, which have applications in nerve regeneration [122]. Myelination is the biological process by which a protective myelin sheath is formed around axons, enhancing the speed and efficiency of electrical signal conduction along nerve fibers [123]. In Figure 5, the diagram shows the structure of a neuron with a myelin sheath and Schwann cells.

The injury can disrupt or inhibit regular mechanisms, resulting in incomplete myelination in damaged areas, which leads to conduction disturbances of nerve impulses. Therefore, it is not surprising that to re-establish cell function, it is necessary to restore myelination. Although research typically prioritizes axon regeneration over remyelination, in some instances, such as specific hereditary disorders or toxin-induced neuropathies, the myelin is primarily damaged, while the axons remain relatively intact, at least in the initial phases of the degeneration [122]. Thus, as the level of knowledge about the specificity of cellular impairment increases, the need to analyze remyelination dynamics becomes emphasized, as it is crucial for the development of effective therapy.

**Figure 6:**
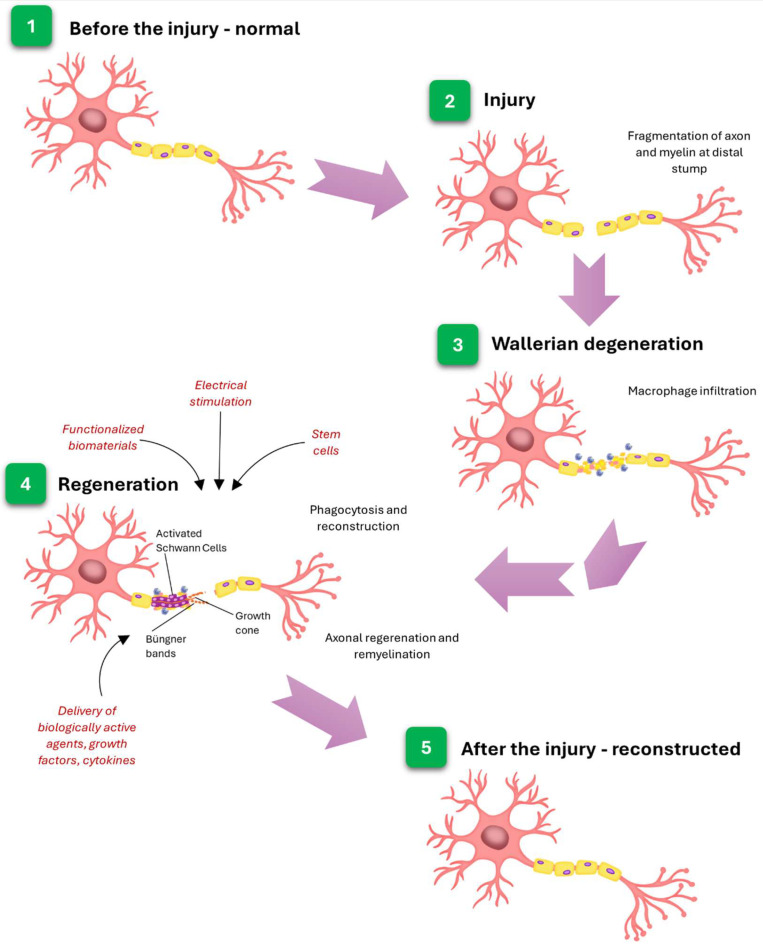
Nerve regeneration after the injury.

Interestingly, not all types of cells exhibit the same capacity for remyelination. For example, peripheral nerves, in contrast to central nervous system cells, are not only characterized by their excellent regeneration potential but also by the extraordinary ability to remyelinate. It is worth noting that myelination in the peripheral nervous system (PNS) is primarily carried out by Schwann cells, which originate from neural crest cells. Moreover, Schwann cells differentiate into either myelinating or non-myelinating types, with each myelinating Schwann cell enveloping a single axon segment. This one-to-one relationship is key for the proper functioning and maintenance of peripheral nerves [122]. Besides transdifferentiating into a repair phenotype promoting axonal regrowth [124], Schwann cells’ role after nerve injury also includes clearing degenerated myelin and axonal debris (during such processes as Wallerian degeneration) and axon remyelination [125]. However, the regenerated myelin sheaths are typically shorter and thinner than the original [126]. In Figure 6, a schematic representation of nerve degeneration and regeneration after the injury is presented.

While Schwann cells are crucial in nerve regeneration, their proper functioning requires intercellular interactions with endothelial cells and fibroblasts, which support the guidance of axons and Schwann cells across damaged sites [127].

Several approaches are being explored to augment the remyelination capacity of Schwann cells. Among these, gene- and cell-based therapies, pharmacological interventions (for instance, agents such as ascorbic acid have been demonstrated to facilitate myelination by supporting basal lamina formation [128]), and the application of biomaterial scaffolds can be listed. In the case of gene therapies, scientists target specific genetic mutations (e.g., the PMP22 gene, responsible for encoding peripheral myelin protein 22, which is critically implicated in the pathogenesis of Charcot-Marie-Tooth disease [129]) to correct aberrant protein expression. A different approach is to replace damaged Schwann cells via transplantation. Biomaterial scaffolds play a crucial role in the process, as they provide structural support for the transplanted cells, enhance their survival, and facilitate integration [130]. Nanoparticles, engineered to mimic the natural environment of the extracellular matrix, can act as scaffolds and directional cues, creating conditions that facilitate Schwann cells’ ability to rebuild the insulating myelin layers essential for nerve function [131]. They might also be used as agents providing cellular alignment in a 3D environment, such as superparamagnetic oleic acid-coated iron oxide nanoparticles (SPIONs) incorporated into poly-l-lactic acid electrospun fiber scaffolds, which were employed to receive oriented guidance for dorsal root ganglion (DRG) neurites [132]. However, the range of nanomaterials in nerve injury applications goes far beyond the ‘typical support’ role. Zong and coworkers presented an interesting strategy: they developed a biological conduit composed of neurotrophin-3-transfected Schwann cells combined with poly(lactic-co-glycolic acid) (PLGA) copolymer and transplanted it into rats. Nanoparticle liposomes were used as a neurotrophin-3 carrier in that work. The study results show that the designed system effectively enhances sciatic nerve regeneration and decreases motor neuron apoptosis [133]. Some other examples of the use of nanomaterials in promoting axonal growth and myelination for nerve regeneration are shown in Table 1.

#### 3.2.3. NPs in Neutrophic/Therapeutic Factors Delivery

Undoubtedly, nanoparticles are most notably recognized for their drug delivery capabilities, a property that is also extensively exploited in the treatment of neurodegenerative diseases [100,134,135,136,137,138]. For instance, NPs are highly effective carriers of neuroactive factors due to their ability to encapsulate these agents. Moreover, after functionalization, nanoparticles can cross biological barriers and deliver therapeutic agents precisely to injury sites, minimizing off-target effects [139,140,141,142,143,144,145,146]. In their extensive publication, Zha and coworkers compiled nanoparticle-based platforms for drug delivery that can cross the blood–brain barrier (BBB) through various transport mechanisms, including diffusion, carrier-, receptor-, or absorptive-mediated transcytosis, efflux pump, and osmosis, which are applied in the treatment of neurodegenerative diseases and cancer therapies. Authors devote considerable space to discussing functionalized polymeric, lipid, and metal nanomaterials that cross the BBB. Such materials are exceptionally suitable for the role thanks to their unique physicochemical characteristics and high biocompatibility [144]. As a result, they have attracted considerable attention within the scientific research community.

An example is the assembled BBB-crossing lipid nanoparticles, which were applied to enable mRNA delivery to neurons and astrocytes in broad brain regions [147]. On the contrary, one non-standard approach to the subject is the engagement of microorganisms. Sun’s team developed the so-called ‘Trojan bacteria’—a drug delivery system based on bacteria (attenuated *Salmonella* strain VNP20009 and *Escherichia coli* 25922 were tested) loaded with glucose polymer (GC) and photosensitive indocyanine green (ICG)-silicon nanoparticles (GP-ICG-SiNPs) aimed at glioblastoma (GBM) photothermal immunotherapy. The studies on the orthotopic GBM mouse model have proven that the platform can bypass the BBB and penetrate cancer tissues, making them susceptible to treatment. Additionally, scientists observed that the designed system promotes innate immune responses due to the tumor-associated antigens (TAAs) released in response to applied irradiation [148].

On the other hand, nanoparticles provide sustained and controlled release of therapeutics, reducing the need for frequent dosing and maintaining effective drug concentrations at the injury site [135,149]. Lu and coworkers proposed a ligand-mediated drug/gene co-delivery platform for glioblastoma multiforme, which allows for controlled and sustained pH-triggered release of therapeutics. Co-encapsulating therapeutic agents within a thermosensitive chitosan-g-poly(N-isopropylacrylamide) (CPN) hydrogel matrix. Specifically, stomatin-like protein 2 (SLP2) short hairpin RNA (shRNA) was combined with irinotecan (CPT-11) and loaded onto cetuximab (CET)-conjugated graphene oxide (GO-CET/CPT11), resulting in the formulation of a composite hydrogel designated as CPN@GO-CET/CPT11@shRNA. The effectiveness of the construct was proven both in vitro and in vivo (with a xenograft of U87 tumor-bearing nude mice). The results not only demonstrate the system’s effectiveness in extended controlled drug release but also highlight its potential for broader applications [150].

Finally, due to the functionalization, nanoparticles demonstrate suitability for localized administration, offering improved selectivity while minimizing off-target effects [151,152,153]. Bai and coworkers presented phosphatidylserine (PS)– and transferrin (TF)-modified liposomes encapsulating dexamethasone (DSS), designated as TF/PS/DSS-LPs, to enhance the treatment of ischemic stroke. Initially, TF molecules targeted the overexpressed transferrin receptor (TfR) in the blood–brain barrier. Upon entry into the brain, the PS modification enabled the liposomes to bind to phosphatidylserine-specific receptors (PSRs) on the surfaces of microglia and astrocytes. This modification also facilitated the uptake of TF/PS/DSS-LPs by these cells. Additionally, it promoted the polarization of astrocytes from the A1 to A2 phenotype and microglia from the M1 to M2 phenotype, thereby decreasing neuronal inflammation and boosting cerebral ischemic injury [154].

Metal-based nanoparticles, particularly iron oxide and gold nanoparticles (AuNPs), represent a prominent class of nanomaterials that have been extensively explored for targeted drug delivery in the treatment of neurological disorders [155,156,157,158]. A recent study by Choi et al. introduces a transferrin-conjugated nanoparticle-based system designed to enhance delivery specificity to brain regions affected by amyloid aggregation in Alzheimer’s disease. The platform, termed Tf-MeLioNs—comprising melittin and L-arginine–coated iron oxide nanoparticles—demonstrated reduced cytotoxicity and hemolytic activity in vitro. Moreover, in vivo experiments using 5XFAD transgenic mice revealed a significant reduction in amyloid plaque burden, particularly within the hippocampal region [159]. In a complementary approach, another research group synthesized AuNPs engineered for the selective targeting of tight junctions and showed that transcranial picosecond laser stimulation, following intravenous administration, significantly enhances blood–brain barrier permeability.

Additionally, the applied modulation is dependent on laser intensity, reversible in nature, and promotes enhanced paracellular diffusion without impairing vasomotion or the structural integrity of the neurovascular unit. The method facilitates the trans-BBB delivery of viral vectors, immunoglobulins, and liposome-encapsulated therapeutics, presenting a promising nanotechnological strategy for drug delivery to light-accessible brain regions and expanding the scope for CNS drug screening and therapeutic applications [160]. Some other examples of the use of nanomaterials in the context of neutrophic factor delivery are presented in Table 1.

Despite the encouraging outcomes associated with targeted therapies utilizing metallic nanoparticles, their clinical application remains constrained by concerns regarding potential toxicity. To address challenges related to bioavailability, a widely adopted strategy involves encapsulating these nanoparticles within biocompatible materials, such as polyelectrolytes. This approach not only improves biocompatibility and stability but also enables the functionalization of the nanoparticle surface with targeting ligands, thereby enhancing the precision of both imaging and therapeutic interventions.

## 4. Polyelectrolyte Materials

Among the diverse classes of biomaterials designed for cell interaction, polyelectrolytes (PEs) have emerged as up-and-coming candidates. Polyelectrolyte-based biomaterials have been employed for several decades across diverse industries, including pharmaceuticals, chemical processing, and food technology. Furthermore, they are employed in biomedicine for tissue engineering, bioimaging, biosensing, and drug delivery [161,162]. Polyelectrolytes participate in delivering regulatory factors, thereby facilitating cell functioning and tissue development, as well as stimulating processes such as differentiation, proliferation, and adhesion [163]. Another beneficial application is the use of polyelectrolytes as delivery systems for therapeutic agents (e.g., drugs and proteins) [164,165,166], as antibacterial factors [167,168,169,170], or in gene delivery systems [171,172,173].

Polyelectrolyte (PE) gels and complexes have gained considerable attention due to their potential to facilitate direct cell contact. Notably, the presence of ionic groups within PEs promotes the formation of stable aqueous dispersions, wherein the ionized polymer chains function as surface-active centers.

The immobilization of cells within polyelectrolyte-based biomaterials has garnered substantial interest in the field of biomedical engineering, owing to its capacity to enhance the regeneration of damaged tissues through the localized production of biologically active molecules, including cytokines and growth factors, which are often deficient or absent due to pathological dysfunction.

**Figure 7:**
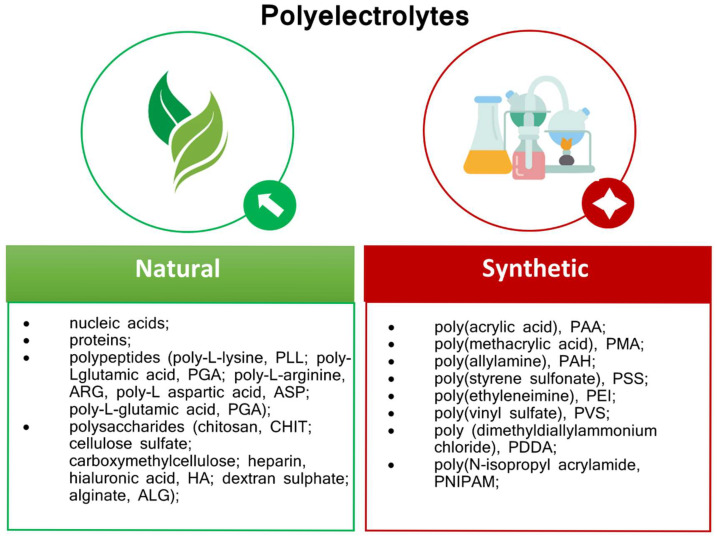
Polyelectrolytes classification.

Considering the origin, two classes of polyelectrolytes are distinguished—natural and synthetic ones (Figure 7). Natural polyelectrolytes include polysaccharides (e.g., sodium alginate (ALG), chitosan (CHIT), hyaluronic acid (HA), cellulose sulfate, heparin, dextran sulfate, carboxymethylcellulose, proteins, polypeptides (like poly-l-aspartic acid (ASP), poly-l-glutamic acid (PGA), poly-l-lysine (PLL), poly-l-arginine (ARG), and nucleic acids [162,166]. On the contrary, among the synthetic polyelectrolytes poly(styrene sulfonate) (PSS), poly(allylamine) (PAH), poly(acrylic acid) (PAA), poly(methacrylic acid) (PMA), poly(ethyleneimine) (PEI), poly(N-isopropyl acrylamide (PNIPAM), poly(dimethyldiallylammonium chloride) (PDDA) and poly(vinyl sulfate) (PVS) can be enumerated [174].

### 4.1. Mechanism of Self-Assembly

Layer-by-layer (LbL) self-assembly can be governed by various intermolecular forces, including electrostatic interactions between oppositely charged species [175], van der Waals forces [176,177], hydrogen interactions [178,179,180,181], hydrophobic interactions [178,182], covalent linkages [183], or synergistic combinations thereof. Multilayer formation through combined mechanisms may be initiated via hydrophobic interactions and subsequently stabilized by hydrogen bonding, resulting in the formation of ultrathin membrane layers. Moreover, electrostatic and hydrophobic forces, as well as electrostatic-hydrogen bonding [184], are commonly associated with the self-assembly process [185]. The morphology of individual monolayers is predominantly influenced by the competitive balance between hydrogen interactions and van der Waals forces, with the resulting two-dimensional nanostructure reflecting this equilibrium [186]. Among these, electrostatic interactions are most widely utilized, enabling the sequential adsorption of oppositely charged polyions onto charged substrates to fabricate polyelectrolyte (PE) multilayer films [186,187] with adjustable thickness and surface topography [188]. Covalent bonding is typically reserved for specialized applications, such as cell immobilization, where it facilitates the attachment of polymers to cell surfaces via chemical or enzymatic conjugation [189,190,191,192,193,194]. Additionally, metabolic introduction may be employed. Amphiphilic polymers, such as temperature-sensitive succinylated pullulan-g-oligo(L-lactide) [195], have been demonstrated to mediate hydrophobic conjugation. Experimental evidence suggests that the interaction between ionic and hydrophobic forces is a crucial factor in both guiding self-assembly and modulating the functional characteristics of the resulting bioactive interface [196].

### 4.2. Polyelectrolytes for Neural Cell Regeneration in Terms of Mechanical Properties and Potential

The limitations of newly developed materials primarily arise from the requirement that biomaterial design be tailored to the specific biological parameters of the target cell types with which they are intended to interact. The example here might be the potential. The action potential of neurons during the depolarization and repolarization cycle (occurring on a time scale of approximately 2 ms) ranges from 30 mV at the transmembrane potential when the neuron is depolarized to a resting potential of −70 mV when the neuron is at rest. In the resting state, the increased number of positively charged sodium ions outside the cell compared to inside creates a concentration gradient. Voltage-gated sodium channels open, leading to the passive transport of positively charged sodium ions from the outside of the cell to the inside, thereby shifting the transmembrane potential toward a positive value. After depolarization, voltage-gated sodium channels close and voltage-gated potassium channels open, causing potassium ions to move from the inside of the cell to the outside, after which the cell repolarizes to its resting potential [197]. Therefore, it can be assumed that the most beneficial would be to work with biomaterials that provide potential values within the range of neuronal function.

The use of polyelectrolytes with various potential values for interaction with neural cells has been reported. Lastly, capsule shells built of poly(allylamine hydrochloride) and poly(4-styrene sulfonate) sodium salt (PAH/PSS) or biodegradable poly-l-arginine and dextran sulfate sodium salt (PArg/DS) with zeta-potential of 3-bilayered samples meanly −18.2 mV and −16.3 mV, respectively, were applied for enhance electrostatic interactions with the plasma membrane, potentially facilitating cellular uptake by neuroblastoma N2A cells [198]. Moreover, polyethylenimine-poly-l-lysine PEI/PLL bilayers, which involve mainly hydrogen interactions between layers, with a positive potential of both layers, mainly +14.9 mV, and +14.1 mV, respectively; furthermore, alginate-chitosan bilayers, with a potential meanly −37.60 and +27.44 [mV], respectively, were assessed for cooperation with cells isolated from embryonic (E19) Wistar rat brains. Evaluation indicates the importance of the potential of the material-cell interface itself, and not the entire membrane system [199].

Conversely, the mechanical properties of biomaterial scaffolds can alter the function of neural cells. Evidence suggested that the soft materials (0.1–1 kPa) promote the differentiation of neurons, whereas the stiffer ones (7–10 kPa) promote the glial differentiation [200,201]. Hydrogels represent a widely utilized class of biomaterials in the treatment of neural dysfunction, mainly due to their controllable mechanical properties. These properties can be precisely modulated by varying the concentration of polymer precursors during hydrogel preparation, a crucial feature for potential clinical applications [202,203].

### 4.3. Polyelectrolytes for Interface with Neural Cells

Several studies have been conducted to explore the usage of polyelectrolytes and their combinations in conjunction with neural cells. As mentioned above, for neuronal cell culture, the commonly applied supports are poly-l-lysine (PLL) and poly-d-lysine (PDL) [16,17,19,204], which are typically applied as monolayers. Poly-d-lysine and poly-l-lysine, the enantiomeric forms of synthetic polylysine, are positively charged molecules that enhance electrostatic interactions with the negatively charged components of the cell membrane. Regarding the charge-related properties of these molecules, both are the same; however, there are reports that poly-l-lysine is subject to deterioration due to the action of proteases released by certain types of cells. Nevertheless, polymers such as HA [205,206,207,208,209], PAH, PAA, and PSS [210] have been identified as potential candidates for such applications. Dąbkowska and co-workers [211] utilized positively charged poly(diallyldimethylammonium chloride) (PDADMAC) and polyanion heparin sodium salt (HP) in their research to construct a polyelectrolyte membrane (PEM) scaffold for the adsorption of brain-derived neurotrophic factor (BDNF). The PEM scaffolds were fabricated using the layer-by-layer (LbL) technique, which relies on the successive deposition of polyelectrolytes with opposite charges. The impact of Brain-derived neurotrophic factor (BDNF) immobilized within poly(diallyldimethylammonium chloride)/heparin PEMs was studied on the SH-SY5Y human neuroblastoma cell line. The SH-SY5Y cell line is heterogeneous, consisting of S-type (epithelial-like) and N-type (neuroblastic) undifferentiated cells. Studies have demonstrated that multilayers terminated with PDADMAC/HP may facilitate the formation of spheroid-like three-dimensional (3D) cell cultures, presenting a promising platform for future investigations as an in vitro model of neurodegenerative diseases. Moreover, polymers such as CHIT, ALG, PEI, and PLL were used to construct multilayer films to examine their interaction with rat neural cells [199]. PEMs multilayer scaffold constructed using the LbL technique, built of PLL as a polycation and poly-l-glutamine acid (PLGA) as a polyanion, was reported by Lee and Wu [212] to promote the differentiation of neural stem/progenitor cells (NSPCs) into neurons with synaptic functions. Those studies showed that NSPCs cultured within PEMs scaffolds without adding serum and growth factors could differentiate into functional neurons with significant neurite outgrowth. NSPCs are multipotent, self-renewing cells that can differentiate into the three principal neural lineages: astrocytes, oligodendrocytes, and neurons. Cerebral cortical NSPCs isolated from 14– to 15–day–old Wistar rat embryos were used to study the upregulation of the number of differentiated neurons and synaptic function with a PLL terminal layer. Some other studies reported the impact of PEMs scaffolds on neural stem/progenitor cells (NSPCs) derived from Wistar rat embryos on days 14–15 [213]. PEMs applied in the research were composed of cationic PAH and anionic PSS or PAA. It was observed that the PAH/PSS multilayer scaffolds stimulate extensive outgrowth and differentiation of NSPCs, primarily into neurons. The neurospheres were unable to attach and differentiate on the PAA-ended multilayers. That work indicates the NSPCs’ dependence on the surface charge of PEM films.

However, the above-mentioned materials are not among the FDA- and/or CE-approved natural and synthetic materials used in clinical trials as components of DDS systems or as catheters for the delivery of cells intended to produce CNTF. To date, nerve conduits, wraps, and cuffs that have received FDA or CE approval are collagen or polyester—based, like RevolNerv^®^ (Orthomed S.A.S., Lyon, France); NeuraGen^®^ and NeuraWrap™ (Integra Life Sciences Corp., Plainsboro, NJ, USA); NeuroMatrix™, Neuroflex™ and NeuroMend™ (Collagen Matrix, Inc., Franklin Lakes, NJ, USA) or Neurolac^®^ nerve guidance (Polyganics B.V., Groningen, The Netherlands) and Neurotube^®^ nerve guidance (Synovis Micro Companies Alliance, Birmingham, AL, USA), respectively [214,215].

## 5. Application of PE and PE-Based Nanocomposites for Neuronal Cell Immobilization

It is noteworthy that, among various scaffolding materials, polymeric hydrogel scaffolds are employed owing to their biocompatibility and structural resemblance to tissue [216,217]; however, they are distinguished by low mechanical strength and limited biological activity. Thus, investigations have been conducted on materials with enhanced physicochemical properties and/or bioactive features. In scaffolds made of polyelectrolyte membranes, various substances can be incorporated, including anticancer factors [218], DNA [219], graphene [220,221], inorganic materials [222], drugs [223], and proteins [224], as well as different types of nanomaterials.

There has been a marked increase in research activity concerning the incorporation of metallic nanoparticles into biomaterial systems. Metal-based NPs employed as additives allow nanocomposites to produce improved mechanical strength, involving additional biological features like antibacterial and antiviral activity [225,226]. Bacteriostatic materials for use in conjunction with neuronal cells have been continually developed.

Electrical stimulation (ES) is a widely discussed strategy for treating nervous tissue. The principle of ES involves the application of electrical impulses to activate neural cells, which are inherently responsive to electrical signals. The demonstrated efficacy of electrical stimulation in modulating neural cell activity and enhancing tissue regeneration presents promising implications for clinical applications. Thus, conductive materials like graphene, carbon nanotubes, silk fibroin-based biomaterials, and metallic nanoparticles can be considered. Besides metallic nanoparticles, various polymers are used as conductive scaffolds, including HA, ALG, poly(pyrrole) (Ppy), polyaniline (PANI), poly(3,4-ethylene dioxythiophene) (PEDOT), and PSS, which are also utilized as components of PEM construction [227,228,229,230].

It can be noted, that those conductive materials for nerves bridging in case of peripheral nerve injury with minor defects have the form of nerve guidance conduits (NGCs) that can mimic the conductivity of natural nerves or and can be applied with electrical stimulation, e.g., the conductive NGCs in form of fiber formed by coprocessing the conductive polymers PEDOT/PSS and CHIT via electrospinning technology [231].

The integration of these two approaches—facilitating the directed differentiation of nerve cells on polyelectrolyte substrates and applying electrostimulation—could efficiently promote nerve tissue reconstruction and advance the elucidation of nerve cell regeneration mechanisms. Furthermore, metallic nanoparticles not only support electrostimulation but also serve as effective antimicrobial agents or drug delivery factors.

## 6. Discussion

Repairing the nervous system is a constant challenge for modern medicine. Research is focused on both cell therapies and biomaterials, as well as their associated technologies. The goal in the latter case is to obtain materials that influence the function, growth, and differentiation of neural cells.

The effectiveness of a material system depends on translational barriers; hence, its design depends on the intended mode of action, such as triggering and enhancing nerve regeneration, and stimulating/supporting neurogenesis directly at the injured site.

In general, it is challenging to determine which of the presented material compositions is best, especially since the effect may be quite the opposite of that achieved with individual material substrates.

As for polyelectrolyte materials, except for material based on chitosan (Reaxon Direct, Kerimedical, Geneva, Switzerland), FDA-approved for nerve gaps bridging [232], none have yet been approved by the FDA and/or CE for use in clinical trials as components of DDS systems or as catheters for the delivery of cells intended to produce CNTF.

Additionally, there are discrepancies in the performance of the substrates in individual experiments, as illustrated by the examples of metallic nanoparticles. For example, CuNPs alone, when analyzed in terms of their toxic effects on zebrafish (Danio rerio) embryos, were reported to induce alterations in the transcriptional expression of genes related to the GABA signaling pathway (gabra1, gad, abat, and gat1). Moreover, CuNPs triggered oxidative stress, as evidenced by disruptions in the activity of dismutase, catalase, and glutathione peroxidase, and elevated malondialdehyde levels. Furthermore, the increase in transcriptional expression of inflammatory markers (IL-1β, TNF-α, IL-6, and IL-8) in the proinflammatory cascade indicated the presence of oxidative stress [233].

On the other hand, some authors assessing on rodent model the neurodegenerative effect of CuNPs on the levels of markers depicting the neurodegenerative changes in the brain observed a reduction in the level of glycosylated acetylcholinesterase, an increase the level of acetylcholinesterase and lipoprotein receptor-related protein 1, a reduction in β-amyloid (βAP) and a reduction in Tau protein indicating neuroprotective effect of CuNPs on rat brain [234].

On the one hand, there are reports that delivery of silver nanoparticles (AgNPs) to microglia may contribute to the reduction in chronic neuroinflammation [235]. On the other hand, AgNPs have been shown to reduce myelin and induce gliosis [236].

At the same time, AgNPs can act as nanocarriers to reduce neurotoxicity. Due to the neurotoxicity associated with the long-term use of tenofovir disoproxil fumarate (TDF), the application of AgNPs-conjugated TDF enabled the achievement of a neuroprotective impact on the hippocampal microanatomy of diabetic rats, reducing the concentration of neuroinflammatory markers (IL-1β and TNF-α) in the hippocampus [237].

Gold nanoparticles are promising due to their biocompatibility, ease of fabrication, and the ability to be functionalized with therapeutic compounds. Subsequent studies confirm the benefits of their use. For example, AuNPs have been reported to be beneficial for human neural stem cells (hNSCs) treated with Amyloid-beta (Aβ), thereby improving mitochondrial function impairment caused by Aβ by elevating mitochondrial membrane potential, upregulating regulators of mitochondrial biogenesis, and inhibiting ROS production. It was found that AuNPs upregulate miR-21-5p expression, thus exerting a cytoprotective effect [238].

Moreover, hNSCs co-treated with AuNPs were observed to be protected from the Aβ-induced reduction in the expression of nuclear factor erythroid 2-related factor 2 (Nrf2) and Nrf2 downstream antioxidant target genes (SOD-1, SOD-2, Gpx1, GSH, Catalase, and HO-1) [239].

It is worth noting that, in the case of carrier systems, the mechanisms of cellular uptake of nanoparticles play a crucial role in their design. Several factors influence interaction with the biological barrier and the uptake of nanocarriers. In addition to the physicochemical properties of the nanocarrier surface, including the charge, its size must also be considered. Typically, the required size of NPs (which may be modified with polyelectrolytes) in drug delivery systems ranges from 10 nm to 200 nm. This size is expected to enable NPs to evade renal clearance, thereby allowing them to persist in the bloodstream for an extended period. However, for delivery to the brain, the optimal size is considered as a balance between BBB penetration and clearance from the bloodstream. Several authors estimated the optimal size of AuNPs delivered to the brain through opened BBB gaps via focused ultrasound-induced BBB opening to be 15 nm [240].

Among metal-based nanoparticles, especially gold nanoparticles, as a representative of this class of nanomaterials, ought to be extensively studied for their use in the treatment of neurological disorders.

However, a thorough assessment of the health risks posed by nanoparticles, due to their persistence in tissues and slow excretion at physiologically relevant concentrations, requires ongoing advanced research.

## 7. Conclusions and Outlook

Repairing the nervous system is a constant challenge for modern medicine. Research is focused on both cell therapies and biomaterials, as well as their associated technologies. The goal in the latter case is to obtain materials that influence the function, growth, and differentiation of neural cells. Polyelectrolyte materials offer some promise in this regard.

Polyelectrolyte layer membrane coatings offer a wide range of possibilities for varying membrane properties, such as thickness, morphology, or wettability, enabling the control of cell culture outgrowth, differentiation, and development.

It is worth noting that layered coating presents some challenges to material stability. The PE materials may undergo deterioration by endocytosis. Also, dissociation from the surrounding medium or body fluids may occur. Thus, only the temporary usability of the systems may be considered. The spatial complexity of neural cells and the optimization of operating time are also factors that limit the usefulness of materials. However, the use of nanotechnology in constructing nanocomposite materials based on polyelectrolytes enables the modification and strengthening of interactions at the material-cell interface.

At the same time, continuous assessment of toxicity or unintended immune reactions is necessary to protect against adverse effects.

We reviewed contemporary strategies for the application of biomaterials in nerve regeneration, with a particular emphasis on nanomaterials that involve nanoparticles and polyelectrolytes. We identify these materials as having significant potential to drive transformative advances in neuroregenerative treatment due to their properties, which allow for achieving a balance between BBB penetration and clearance from the bloodstream.

It can be assumed that future work on systems of polyelectrolyte membrane nanocomposites with immobilized cells will focus on developing functional materials incorporating biological activity to enhance/modulate cell function, as well as novel cell types.

## Figures and Tables

**Table 1:** Key Mechanisms of Neuroprotection/Regeneration.

Action	Description	Examples
Antioxidant and Anti-Inflammatory Actions		
Oxidative Stress Mitigation	NPs can scavenge reactive oxygen species (ROS), protecting neurons from oxidative damage.	Cerium oxide nanoparticles loaded with therapeutic agents protect mitochondria and suppress neuronal apoptosis, allowing for suppression of neuroinflammatory processes in the middle cerebral artery embolization/recanalization mouse model [107]. Zein nanoparticles loaded with chloroquine (Zein-CQ NPs) inhibit neuronal apoptosis, reduce neuroinflammation, and increase M2 phenotype microglia polarization, improving functional nerve recovery after SCI in rats [108]. ROS-responsive quercetin-based polydopamine nanoparticles (Que@DAR NPs) suppressed neuroinflammation, decreased neuronal apoptosis, and markedly improved neurological function in a rat model of middle cerebral artery occlusion (MCAO) [109]. Iron oxide (Fe_3_O_4_) nanoparticles coated with omega-3 (Fe_3_O_4_@ω_3_) enhance structural and functional sciatic nerve regeneration by demonstrating a neuroprotective effect in a rat model [110].
Inflammation Modulation	NPs can decrease proinflammatory cytokines, creating a favorable environment for nerve regeneration.	
Enhancing neuronal viability	NPs can reduce cellular apoptosis.	
Promotion of Axonal Growth and Myelination		
Axonal Regeneration	Surface-modified NPs can guide axonal growth by mimicking extracellular matrix structures.	Conductive topological scaffold—Morpho butterfly wing with reduced graphene oxide (rGO) nanosheets and methacrylate gelatin (GelMA) hydrogel with encapsulated brain-derived neurotrophic factor (BDNF) (rGO/BDNF/GelMA-integrated Morpho butterfly wing) enhances neurite elongation and functional recovery in a rat model [111]. Co-spin polycaprolactone (PCL) and graphene oxide-oriented hybrid nanofibrous scaffold (O-GO/PCL) enhance axonal regeneration, increase remyelination, and elevate functional recovery in rats [112]. NPs delivering neurotrophic factors like BDNF or insulin-like growth factor 1 (IGF-1) have been shown to increase the density of myelinated axons and support muscle preservation post-injury [113,114]. CNM-Au8 (a clean-surfaced, faceted gold nanocrystal (AuNC)) promotes myelin generation in vivo animal models of multiple sclerosis [115]. Selenium-doped carbon quantum dots (Se-CQDs) reduce nerve fibers’ demyelination and neuronal cell apoptosis after traumatic spinal cord injury in rats [116]. Biomimetic collagen gel with magnetic nanoparticles (MNPs) coated with nerve growth factor (NGF) supports axon regeneration by promoting its growth [117].
Myelination Support	NPs can promote axon remyelination.	
Enhanced Drug Delivery		
Targeted Delivery	Functionalized NPs can cross biological barriers and deliver therapeutic agents directly to injury sites, minimizing off-target effects.	Chitosan-halloysite nanotube composite (NGCs) featuring aligned microchannel pores have been designed to enable the sustained release of the neurogenic agent 4-aminopyridine (4-AP), thereby promoting peripheral nerve regeneration in a rat model [118]. Bioactive 3D Scaffolds (Polycaprolactone hollow tubes called nerve guide conduits) supply a sustained release of heparin-bound nerve growth factor (NGF) or brain-derived neurotrophic factor (BDNF) electrostatically immobilized onto an amine-functionalized surface to encourage neurite outgrowth and Schwann cell migration [119]. A nanofibrous scaffold fabricated via emulsion electrospinning of poly(L-lactic acid) (PLLA) has been developed for the controlled delivery of recombinant human nerve growth factor (NGF) and recombinant human vascular endothelial growth factor (VEGF), aiming to facilitate peripheral nerve regeneration [120]. Electrospinning multilayered scaffolds (polycaprolactone nanofibers with magnetic nanoparticles—Fe_3_O_4_) for sustainable release of melatonin for peripheral nerve regeneration [121].
Controlled Release	NPs can provide sustained release of therapeutics, reducing the need for frequent dosing and maintaining effective drug concentrations at the injury site.	

## Data Availability

Not available.
